# Bandgap Engineering and Near-Infrared-II Optical Properties of Monolayer MoS_2_: A First-Principle Study

**DOI:** 10.3389/fchem.2021.700250

**Published:** 2021-06-18

**Authors:** Ke Yang, Tianyu Liu, Xiao-Dong Zhang

**Affiliations:** ^1^ Department of Physics and Center for Joint Quantum Studies, School of Science, Tianjin University, Tianjin, China; ^2^ Tianjin Key Laboratory of Brain Science and Neural Engineering, Academy of Medical Engineering and Translational Medicine, Tianjin University, Tianjin, China

**Keywords:** monolayer MoS_2_, point defects, electronic structure, dielectric function, absorption, near-infrared-II

## Abstract

The fluorescence-based optical imaging in the second near-infrared region (NIR-II, 1,000–1,700 nm) has broad applications in the biomedical field, but it is still difficult to find new NIR-II fluorescence materials in the two dimension. As a crucial characteristic of the electronic structure, the band structure determines the fundamental properties of two-dimensional materials, such as their optical excitations and electronic transportation. Therefore, we calculated the electronic structures and optical properties of different crystalline phases (1T phase and 2H phase) of pure monolayer MoS_2_ films and found that the 1T phase has better absorption and thus better fluorescence in the NIR-II window. However, its poor stability makes the 1T-phase MoS_2_ less useful *in vivo* bioimaging. By introducing vacancy defects and doping with foreign atoms, we successfully tuned the bandgap of the monolayer 2H-MoS_2_ and activated it in the NIR-II. Our results show that by engineering the vacancy defects, the bandgap of the 2H phase can be tailored to around 1 eV, and there are three candidates of vacancy structures that exhibit strong absorption in the NIR-II.

## Introduction

The NIR-II fluorescence probe has attracted much attention in recent years. Compared with the visible light (400–700 nm) and the NIR-I (700–900 nm) imaging, the NIR-II (1,000–1,700 nm) imaging has longer fluorescence wavelength and stronger penetrating ability, which has shown great potential in tumor diagnosis and *in vivo in situ* imaging. Since Dai *et al.* discovered that carbon nanotubes (Jiao et al., 2009; Welsher et al., 2009), fluorescent quantum dots (Hong et al., 2012), small molecules (Antaris et al., 2016), and rare earth metals have good fluorescence and biocompatibility in the NIR-II window (Sun et al., 2019), the development of this technology has been widely promoted (Hong et al., 2012; Fan et al., 2018). With the rapid development of imaging equipment and technology in the NIR-II (Zhang et al., 2016; Liu et al., 2019), finding new fluorescence material in the NIR-II is still a pressing task, which requires us to find semiconductor materials with a bandgap less than 1.0 eV. It is well-known that two-dimensional materials have obvious advantages in bandgap modulation; however, in recent years, there have been very few theoretical works studying the fluorescence materials in the NIR-II. As a result, searching for the well-behaved two-dimensional materials relies more on perceptions and arduous experiments. Among these efforts, layered transition metal dichalcogenides (TMDs) (Soares et al., 2020) have been widely studied in recent years due to their unique atomic structure, band structure, and optical properties (Wang et al., 2012). Thereinto, MoS_2_ with natural bandgap (1.2∼1.8 eV) come to the fore. In the benefit of that, all the TMDs_2_-MX_2_ (M = Mo, W; X = S, Se) possess similar electronic structures and thus similar properties in photoelectric and catalytic processes; MoS_2_ is usually studied as a representative for TMDs (Hong et al., 2017).

**FIGURE 1:**
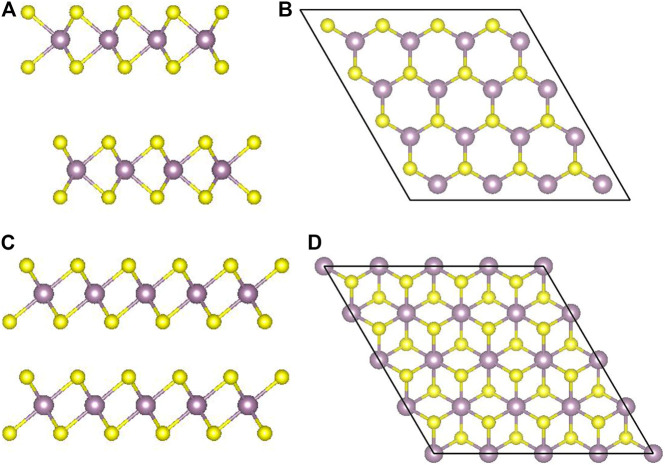
1T- and 2H-phase MoS_2_: **(A)** and **(B)** the layered structure of 2H-MoS_2_ and the top view in z direction; **(C)** and **(D)** the same for 1T-MoS_2_.

MoS_2_ has three types of crystal structures: 1T (trigonal), 2H (hexagonal), and 3R (rhombohedral). The 1T phase is metallic, while the 2H and the 3R are semiconductors. Besides, the 2H phase is the most stable one (Xu et al., 2016). 1T and 3R are metastable and can be converted back to the 2H phase (Chaste et al., 2020) under the conditions of heating and annealing (Geng et al., 2016; Byrley et al., 2019). As shown in Figure 1, a monolayer MoS_2_ film is composed of two layers of sulfur atoms sandwiched by one layer of Mo atoms (Helveg et al., 2000). The atoms of different layers are bonded by strong covalent bonds. The thickness of the monolayer MoS_2_ is about 6.5 Å. The upper and lower S atoms of the 2H phase overlap completely in the direction perpendicular to the film, while the upper and lower S atoms of the 1T phase do not overlap at all.

The layered nature of bulk MoS_2_ makes it easy to slide subjected to external friction (Zhang et al., 2009). Therefore, 2H-MoS_2_ has been widely used in the field of lubricants (Lahouij et al., 2012) and hydrodesulfurization catalysts (Gutiérrez et al., 2014). When reducing to single-layer structures, the bandgap of MoS_2_ increases from ∼1.2 to ∼1.8 eV and changes from indirect bandgap to direct, which indicates that the layered MoS_2_ bandgap is adjustable and has potential applications in optoelectronics (Steinhoff et al., 2015). Because of a direct bandgap of ∼1.8 eV, monolayer MoS_2_ has great applications in photodetectors and plasma devices. Moreover, as an attractive nanomaterial for photothermal cancer therapy, 1T-phase MoS_2_ is a highly effective reagent in photoacoustic imaging (PAI)–guided photothermal therapy (PTT) in the NIR-II window (Zhou et al., 2020). Under 1,064 nm laser irradiation, it can effectively ablate cancer cells *in vitro* and tumors *in vivo*. However, monolayer 1T-MoS_2_ is less stable than the 1H phase. Therefore, it is necessary to find a way to engineer 1H-MoS_2_, so that it can be used for fluorescence in the NIR-II. At present, different point defects and antisite defects (Nakayasu et al., 2019) have been observed experimentally. In the study of Zhou *et al.*, point vacancies are more common than antisite defects, which is consistent with their DFT calculations, and mono-vacancy V_S_ (only one S atom missing from the sulfur sublattice site) has the lowest formation energy. And V_MoS2_ (containing a V_Mo_ and V_S2_ pair), V_MoS3_ (vacancy complex of Mo atom and nearby three S atoms), and V_MoS6_ (vacancy complex of Mo atom nearby three S pairs) have relatively poor structural stability due to its higher formation energy (Zhou et al., 2013). Mann *et al.* studied MoS_2(1-x)_ Se_2x_ doped with different concentrations of Se and concluded that the bandgap decreases linearly with increasing Se-doping concentration with the tuning window ranging from 1.85 eV (pure MoS_2_) to 1.55 eV (pure MoSe_2_) (Li et al., 2014), which provides a feasible way to manipulate of the bandgap of MoS_2_ (Mann et al., 2014). Dumcenco *et al.* reported single-layer Mo_1-x_W_x_S_2_ semiconductor alloy with a bandgap ranging from 1.82 eV to 1.99 eV, not significantly depending on the concentration of W (Dumcenco et al., 2013). Inspired by previous studies (Zeng et al., 2020), we calculated the electronic structures and optical properties of 1T and 2H phases of monolayer MoS_2_ films by the first-principle method. By adjusting the position of the vacancy defects and dopings, we predict three possible structures that can be applied in the NIR-II, which provides a potential direction for experimentally studying NIR-II fluorescence of single-layer MoS_2_ films.

The article is organized as below. *Theories* reviews basic steps in calculating the optical properties from the first-principal methods. *Results and Discussion* presents our results and discussions on band structures, ground-state energies, and absorption spectra for different defects and dopings. The technique details are listed in Methods.

## Theories

**FIGURE 2:**
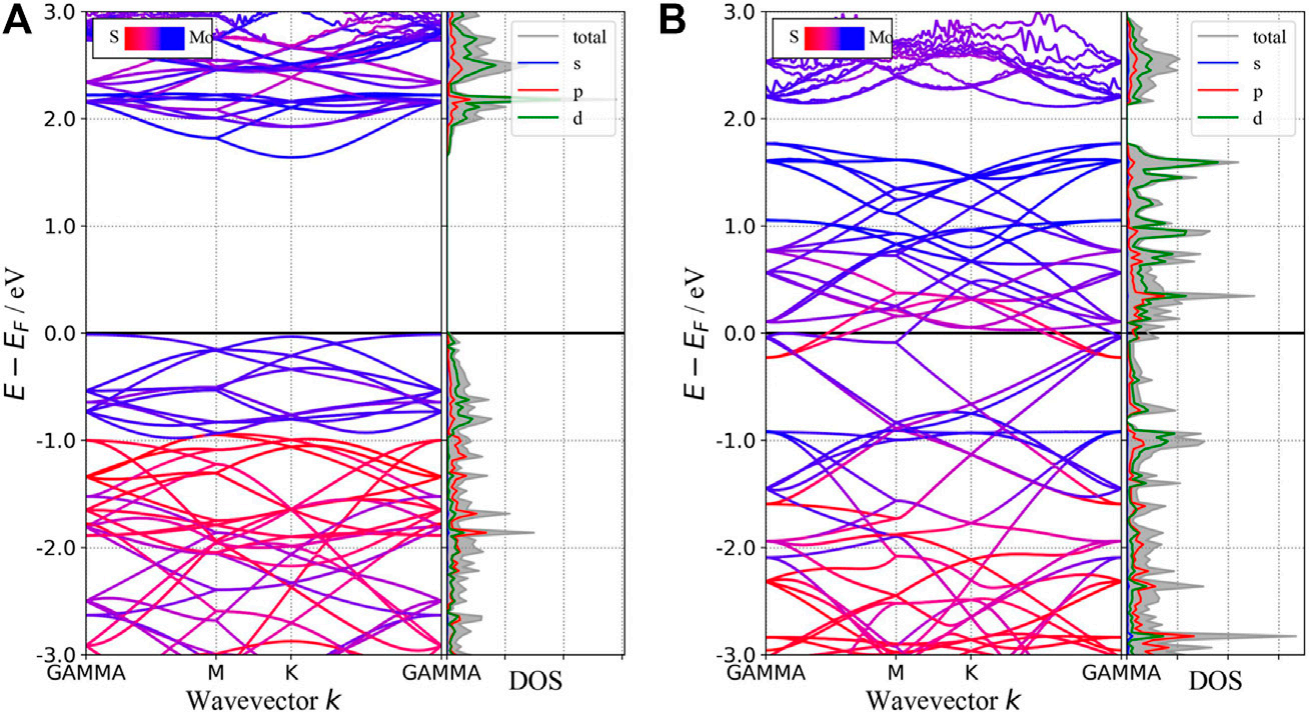
Band structures and pDOS of 2H-MoS_2_ **(A)** and 1T-MoS_2_ **(B)**. The blue (red) curves are mainly contributed by atom Mo (S) in the band structure. The shadow area in the pDOS refers to the total DOS, and the solid lines of different colors refer to the projection on different orbitals indicated by the legend inside the figure.

In this section, we will discuss the basic concepts of how to get the absorption spectra of two-dimension materials from the first-principle calculations. Although one can get the plasma frequency and the dynamic dielectric function from VASP directly and the absorption spectra seem to be obtained straightforwardly from the imaginary part of the dielectric function, special attention should be paid to the metallic phase. According to the band structure as shown in Figure 2B, 1T-phase monolayer MoS_2_ behaves like a metal. Therefore, its dynamic dielectric function consists of two parts: one from the interband transition, which can be obtained from the optical calculation module in VASP, the other from the intraband transition, which requires some extra steps recapitulated as below.

For metals, the optical complex dielectric function consists of interband and Drude-like intraband contributions (Guan et al., 2015):

$$\varepsilon \left(\omega \right)=\varepsilon^{\mathrm{int}ra}\left(\omega \right)+\varepsilon^{\mathrm{int}er}\left(\omega \right).$$ (1)

The imaginary part of the interband dielectric function is given by the first-principle calculations as follows (Gajdoš et al., 2006)

$$\varepsilon_{\alpha \beta }^{\left(2\right)}\left(\omega \right)=\frac{4\pi^{2}e^{2}}{\Omega }\underset{q\to 0}{\lim}\frac{1}{q^{2}}\times \sum_{c,v,\vec{k}}2w_{k}\delta \left(E_{c}-E_{v}-\omega \right){\left|\langle c|\hat{e}\cdot \vec{q}|v\rangle \right|}^{2},$$ (2)

where $\hat{e}$ is the polarization direction of the photon, $\vec{q}$ is the electron momentum operator, and the $\langle c|\hat{e}\cdot \vec{p}|v\rangle$ is the optical transition amplitude from the valence bands (v) to the conduction bands (c). α and β refer to Cartesian coordinates, Ω is the volume of the unit cell, and the integration over $\vec{k}$ is performed by summation over special k-points with a corresponding weighting factor $w_{k}$. The real part of the interband dielectric function $\varepsilon_{1}\left(\omega \right)$ is obtained from the imaginary part $\varepsilon_{2}\left(\omega \right)$ based on the usual Kramers–Kronig transformation,

$$\varepsilon_{1}\left(\omega \right)=1+\frac{2}{\pi }P\int_{\text{0}}^{\infty }\frac{\varepsilon_{\alpha \beta }^{\left(2\right)}\left(\omega \prime \right)\omega \prime }{\omega \prime^{2}-\omega^{2}+i\eta }d\omega \prime ,$$ (3)

where P denotes the principal value and η is the complex shift parameter. The intraband contribution is usually modeled by the Drude model (Olmon et al., 2012):

$$\begin{array}{l} \varepsilon_{1}\left(\omega \right)=1-\frac{\omega_{p}^{2}\tau_{D}^{2}}{1+\omega^{2}\tau_{D}^{2}},\\ \varepsilon_{2}\left(\omega \right)=\frac{\omega_{p}^{2}\tau_{D}}{\omega \left(1+\omega^{2}\tau_{D}^{2}\right)}, \end{array}$$ (4)

where $\tau_{D}$ is electron relaxation time and $\omega_{p}$ is intraband plasma frequency. $\omega_{p,\alpha \beta }$ is the plasma frequency tensor which can be calculated using (Harl, 2008):

$$\omega_{p,\alpha \beta }^{2}=-\frac{4\pi e^{2}}{\Omega }\sum_{nk}2\frac{\partial f\left(\varepsilon_{n}\right)}{\partial \varepsilon_{n}}\left(e_{\alpha }\frac{\partial \varepsilon_{n}\left(k\right)}{\partial k}\right)\left(e_{\beta }\frac{\partial \varepsilon_{n}\left(k\right)}{\partial k}\right).$$ (5)

In this study, we took into account the electron–electron scattering only ($\tau_{D}=\tau_{ee}$) and neglected the other scattering processes, such as those involving phonon, impurity, and defects. After the GW approximation (Shishkin and Kresse, 2006; Shishkin and Kresse, 2007), the inverse of the electron relaxation time (electron relaxation rate) (Ogawa and Petek, 1996) can be obtained from the imaginary part of the electron self-energy Im∑(εj) (Schöne, 2007; Cazzaniga, 2012):

τee−1(j)=2|〈j|Im∑(Ej)|j〉|=2Zj|〈j|Im∑(εj)|j〉|, (6)

where j represents the Kohn–Sham eigenstate, and $Z_{j}$ is the quasi particle correction factor given by GW calculation, $Z_{j}^{-1}=1-\langle j|{\left|\frac{\partial \sum \left(\omega \right)}{\partial \omega }\right|}_{\omega =\varepsilon_{j}}|j\rangle$.

Different from bulk materials, the absorbance of two-dimensional materials is defined by (Wang et al., 2019):

A(ω)=Reσ2D(ω)/ε0c. (7)

The in-plane 2D optical conductivity is directly related to the corresponding $\sigma_{3D}\left(\omega \right)$ component through $\sigma_{2D}\left(\omega \right)=L\sigma_{3D}\left(\omega \right)$, where L is the slab thickness in the simulation cell. Based on the Maxwell’s equations, the 3D optical conductivity can be expressed as below:

$$\sigma_{3D}\left(\omega \right)=i\left[1-\varepsilon \left(\omega \right)\right]\varepsilon_{0}\omega \text{.}$$ (8)

Combining with the above equation, we obtained the absorption spectra A(ω) of two-dimensional materials by $A\left(\omega \right)=\frac{L\omega \varepsilon_{2}}{c}$.

## Results and Discussion

### 1T-MoS_2_ and 2H-MoS_2_

In this subsection, we will compare the band structures and the optical properties of monolayers 1T-MoS_2_ and 2H-MoS_2_ without defects and doping. The band structures and density of states (DOS) of 1T and 2H phases are shown in Figure 2. It shows that monolayer 2H-MoS_2_ remains a semiconductor as the bulk MoS_2_, while monolayer 1T-MoS_2_ turns into a metal, whose states near the Fermi surface mainly come from the Mo–d orbit and the S–p orbit. The 2H-phase monolayer shows a direct bandgap of 1.65 eV, close to the calculated result of Kan *et al.* (1.67 eV) and Johari *et al.* (1.68 eV) (Johari and Shenoy, 2012; Kan et al., 2014). The results are in the same bandgap as GGA-PBE predicts (Komsa and Krasheninnikov, 2015), and this ensures the rationality of the calculated parameters in this study. However, since the DFT-GGA tends to underestimate the bandgap, our result is lower than the experimental value ∼1.8 eV (Kam and Parkinson, 1982; Mak et al., 2010).

**FIGURE 3:**
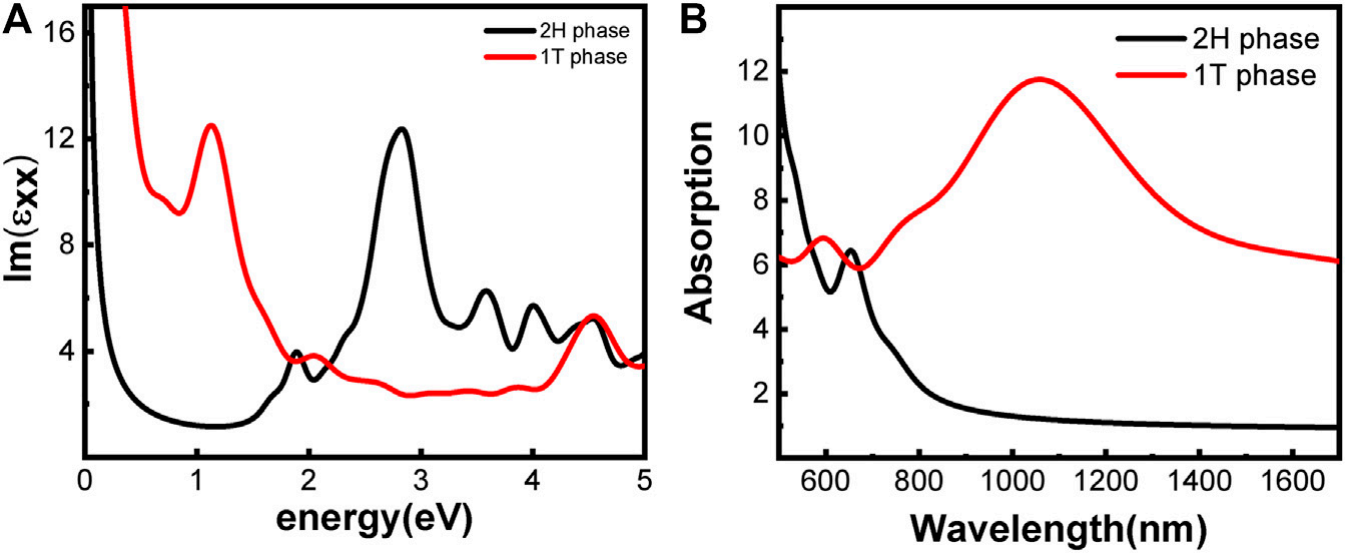
Optical properties of monolayer MoS_2_ without defect and doping: **(A)** imaginary part of the dielectric function; **(B)** absorption spectra. The phases are indicated by color.

The optical properties of the two phases are shown in Figure 3. 2H-MoS_2_ shows peaks at 1.89 eV and 2.82 eV in the imaginary part of the complex dielectric functions (Figure 3A), which is mainly due to the electron transition between the highest valence band Mo–3d orbit and the conduction bands Mo–3d and S–p orbitals, according to the partial density of states (pDOS) as shown in Figure 2. The monolayer 2H-MoS_2_ imaginary part of the dielectric functions peaks at 1.89 eV and matches the lower absorption resonance in both its position and width. Therefore, we attribute the photoluminescence of monolayer MoS_2_ to direct-gap luminescence. These findings are in good agreement with the experimental results of Mak *et al.* (Mak et al., 2010) and previous calculations (Zeng et al., 2020) for monolayer MoS_2_ (Kumar and Ahluwalia, 2012). The transition of the 1T phase occurs at a lower energy of 1.12 eV, corresponding to the Mo–d and S–p transitions. The characteristic peaks in the dielectric functions for each phase lead to the distinct absorption spectra in Figure 3B. The 2H phase mainly absorbs light with a wavelength below 700 nm, while the 1T phase shows a strong absorption in the NIR-II window and an absorption peak at 1050 nm, which is consistent with the experimental conclusion of Zhou *et al.* (Zhou et al., 2020). However, the 1T phase is less stable than the 2H phase. Therefore, it will be useful to find a way to modify the band structure of the 2H phase while keeping its stability. We found that the introduction of vacancy defects or doping of foreign atoms into the 2H phase shows great promise for its application in the NIR-II window. The results will be presented in rest of the article.

### 2H-MoS_2_ With Vacancy and Doping

#### S-Vacancy Structures

**FIGURE 4:**
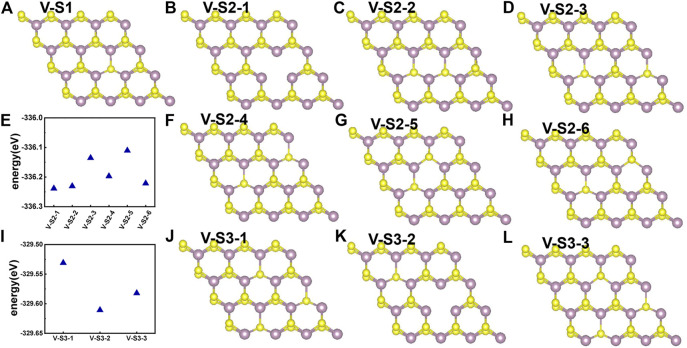
Different S-vacancy defect structures of 2H-MoS_2_, rotated by an angle to distinguish the two S layers: **(A)** single S-vacancy; **(B–D)** and **(F–H)** six types of configuration for double S-vacancies; **(J–L)** three types of configuration for triple S-vacancies; **(E)** and **(I)** compares the ground-state energy of double and triple S-vacancies.

**FIGURE 5:**
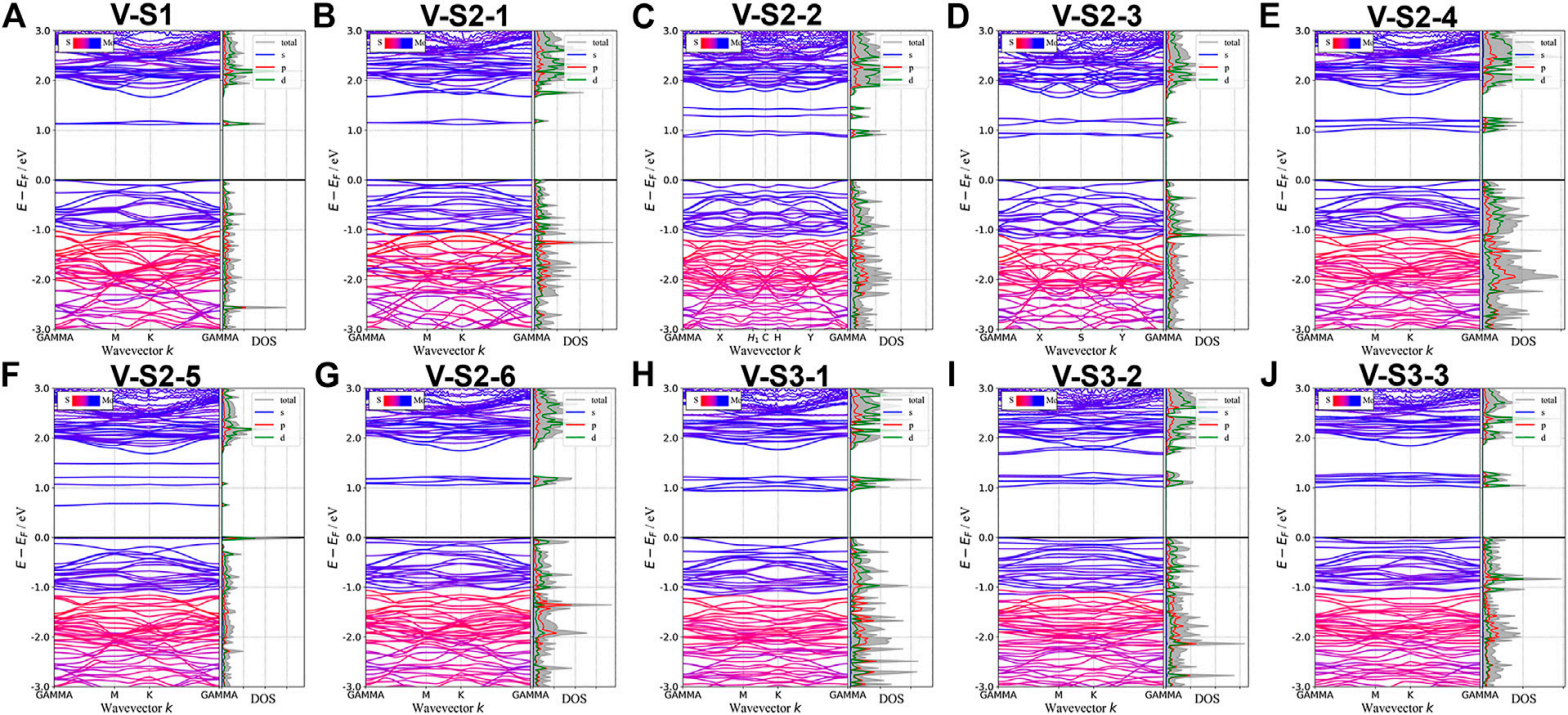
Band structures and pDOS of monolayer 2H-MoS_2_ with different S-vacancy defects. The labels of different vacancy structures are denoted above each figure. The corresponding atomic configurations can be referred to Figure 4.

Point defects in monolayer MoS_2_ are attractive because defects play a crucial role in manipulating its electrical and optical properties. Therefore, we designed the supercell of 2H-MoS_2_ with different numbers of S-vacancy, whose configurations have been plotted in Figure 4. Figure 4A shows single S-vacancy structure, and Figures 4C,D,F show two S-vacancies in the same atomic plane. Figures 4B,G,H show two S-vacancy structures in different atomic planes. Figure 4E compares the ground-state energy of the six configurations of double S-vacancies, demonstrating the structure stability, indicating that configurations V-S2-1, V-S2-2, and V-S2-6 are relatively stable, compared to V-S2-3, V-S2-4, and V-S2-5. Figure 4J shows triple S-vacancy structures in the same plane. Figures 4K,L show the triple S-vacancy structures in different planes. Figure 4I compares the ground-state energy of the three configurations of triple S-vacancies and indicates configuration V-S3-2 is the most stable among the three, while V-S3-1 is the worst. To explore the influence of S-vacancy on the electronic structure of the system, we calculated the band and pDOS, as shown in Figure 5, and found that different S-vacancy defects induce different energy levels contributed by Mo–d and S–p in the bandgap relative to the pure MoS_2_, which are distributed around 1 eV above the Fermi energy. In addition, it is noted that the density of states of the defect states in the bandgap increases with the increasing number of vacancies. The introduction of vacancies significantly reduces the bandgap. We also summarized the width of bandgap and the number of vacancies in Table 1 but found there is no direct relation between the two.

**TABLE 1:** Bandgap of different S-vacancy model.

S-vacancy model	Bandgap (eV)	S-vacancy model	Bandgap (eV)
V-S1	1.124	V-S2-5	0.657
V-S2-1	1.115	V-S2-6	1.073
V-S2-2	0.879	V-S3-1	0.950
V-S2-3	0.864	V-S3-2	1.031
V-S2-4	0.975	V-S3-3	1.016

**FIGURE 6:**
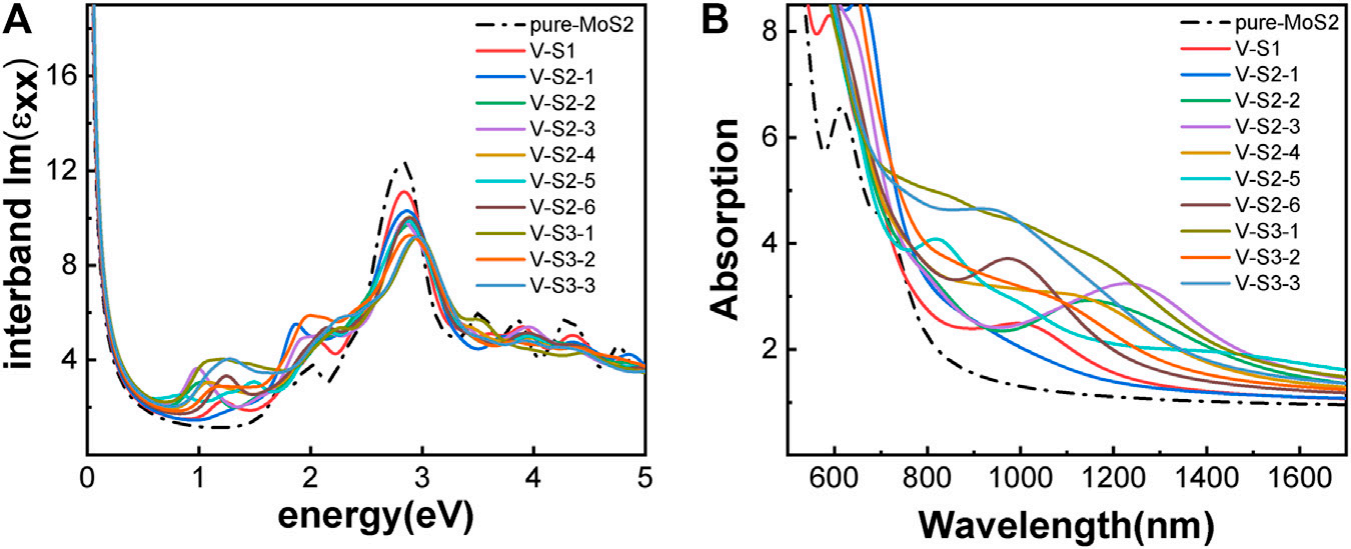
Optical properties of monolayer 2H-MoS_2_ with different S-vacancy defects: **(A)** the imaginary part of the dynamic dielectric function; **(B)** absorption spectra. The black dotted line is for pure monolayer 2H-MoS_2_. The labels in the legend refer to different vacancy structures as shown in Figure 4.

Next, we studied the effects of S-vacancies on the optical properties. The imaginary part of the dielectric function is highly related to the absorption coefficient of the material. As shown in Figure 6A, in the presence of S-vacancy, the imaginary part of the dielectric function appears at a transition peak around 1 eV due to the energy levels between the bandgap of pure MoS_2_ (Figure 5), but the transition peak around 1 eV is lower than the peak at the higher energy. Rai *et al.* studied the optical properties of the S-vacancy concentration from 0 to 50% (0, 12.5, 25, 37.5, and 50%) and found a transition peak at a lower energy range, which would significantly increase with the increasing vacancy concentration (Rai et al., 2020). However, our calculations show that as the number of vacancies increases, the stability of the structure becomes worse. Although the density of defect states is small, it enhances the absorption in the NIR-II window significantly, as shown in Figure 6B. Taken together, the stability of the ground-state energy and absorbance, configurations V-S2-2, V-S2-3, and V-S2-6 are the best whose peaks are at 1,150 nm, 1,240 nm, and 980 nm, respectively.

#### Mo-Vacancy Structures

**FIGURE 7:**
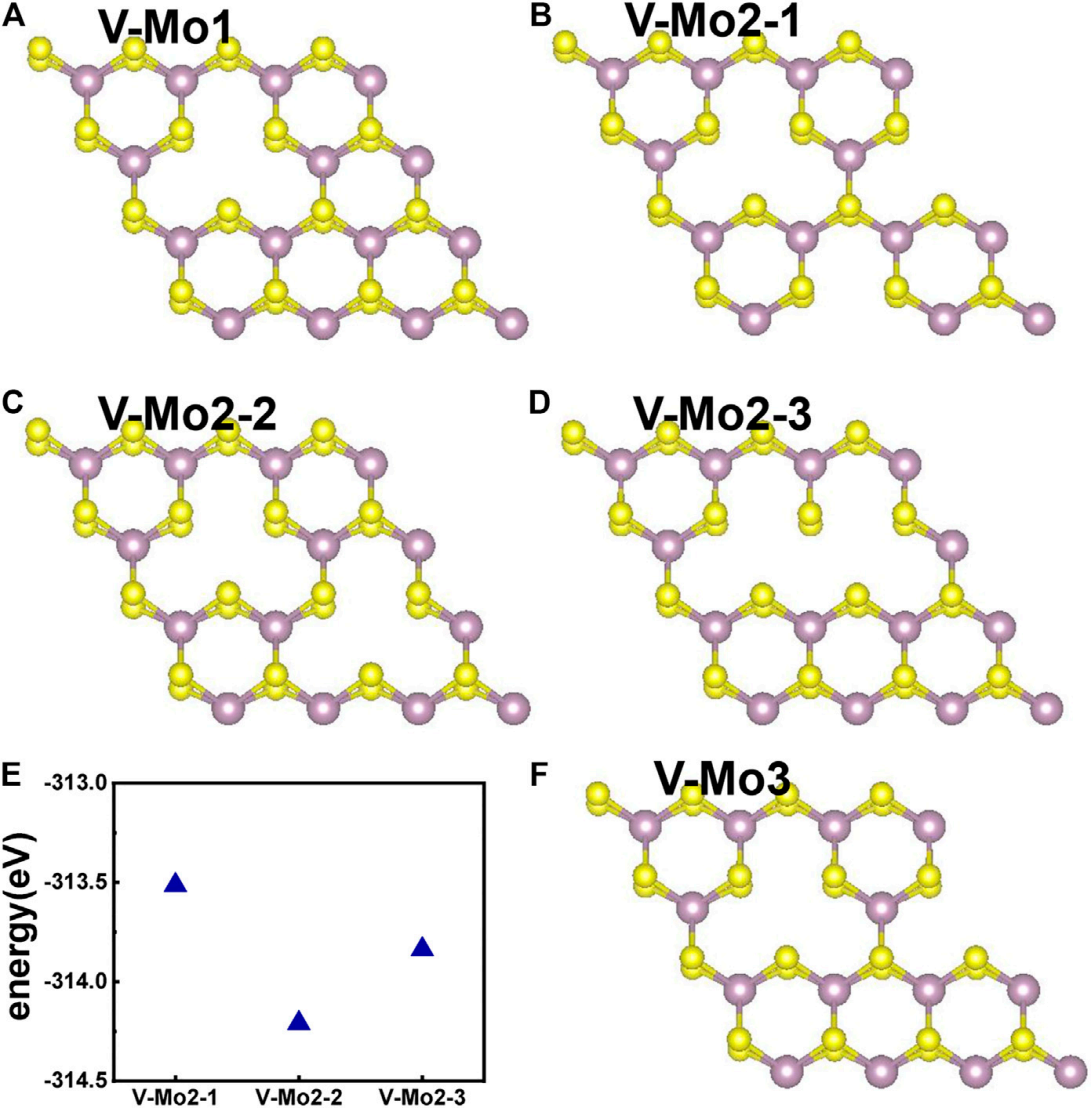
Different Mo-vacancy defect structures of 2H-MoS_2_, rotated by an angle to distinguish the two S layers: **(A)** single Mo-vacancy; **(B–D)** three configurations of double Mo-vacancies; **(F)** one configuration of triple Mo-vacancies; **(E)** comparison of the ground-state energies of different configurations of double Mo-vacancies.

**FIGURE 8:**
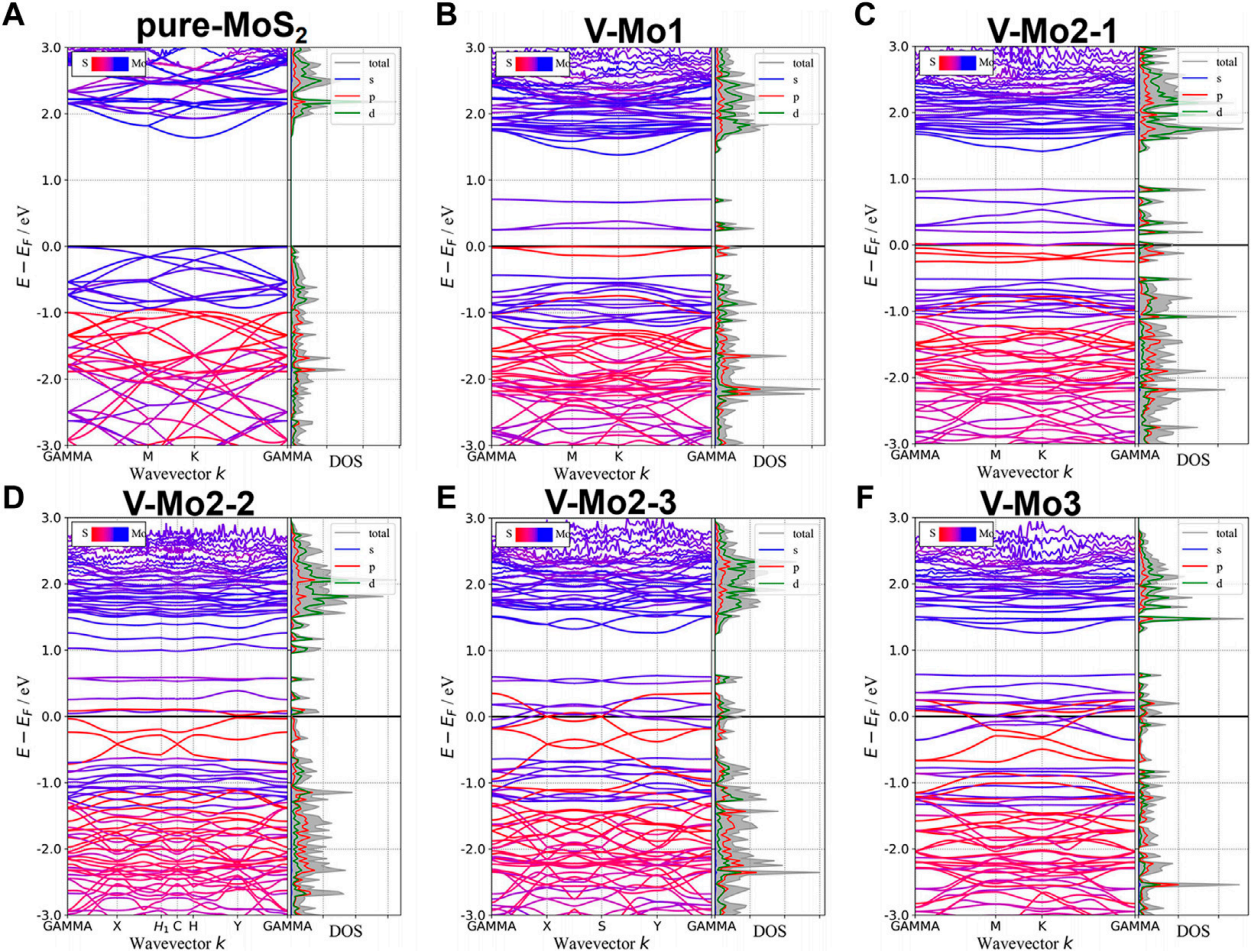
Band structures and pDOS of monolayer 2H-MoS_2_ with different Mo-vacancy defects. The labels of different vacancy structures are denoted above each figure. The corresponding atomic configurations can be referred to Figure 7.

**FIGURE 9:**
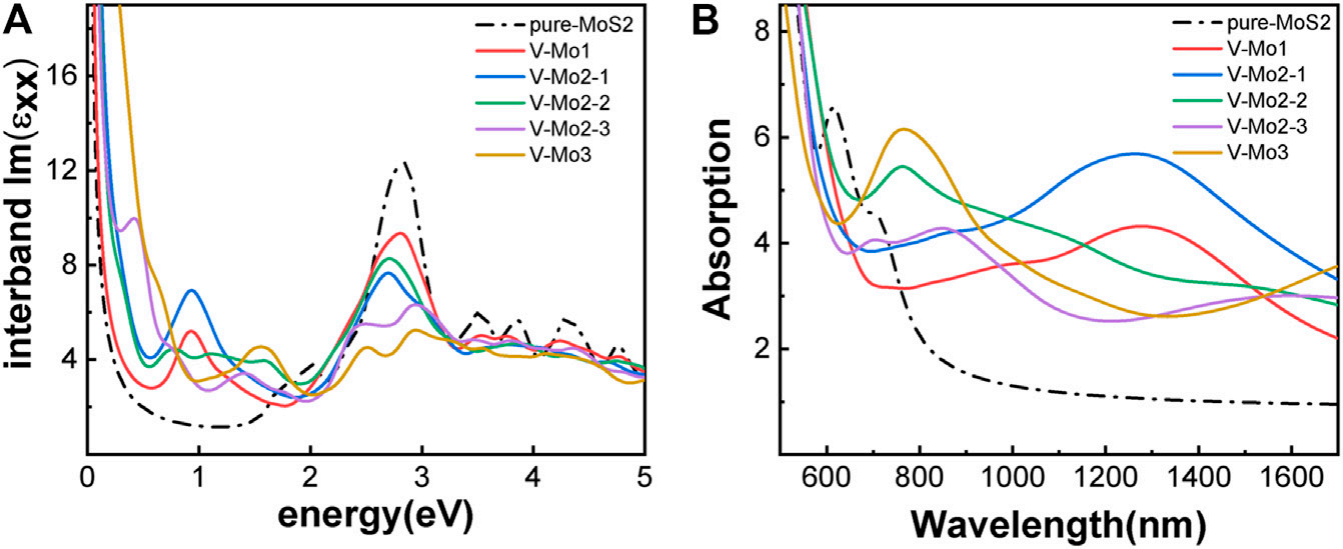
Optical properties of monolayer 2H-MoS_2_ with different Mo-vacancy defects: **(A)** the imaginary part of the dynamic dielectric function; **(B)** absorption spectra. The black dotted line is for pure monolayer 2H-MoS_2_. The labels in the legend refer to different vacancy structures, as shown in Figure 7.

Figures 7A–D,F give the different Mo-vacancy structures. In analogy to the S-vacancy structures, we considered the configurations of single, double, and triple Mo-vacancies. Judging from the ground-state energy, as shown in Figure 7E, V-Mo2-2 is the most stable among the configurations of double Mo-vacancies. With introducing Mo-vacancy, monolayer 2H-MoS_2_ tends to be a metal; as shown in Figure 8, except for the configurations of V-Mo1 and V-Mo2-2 whose bandgaps are 0.26 eV and 0.04 eV, respectively. The occupied states at the Fermi level are mainly contributed by the S–p and Mo–d orbitals. Figure 9 shows the dynamic dielectric function and the absorption spectra of the series of configurations of Mo-vacancy. It can be seen from the imaginary part of the dielectric function that the peak at 2.81 eV is quenching with the increasing number of Mo-vacancy, which is consistent with the band structure analysis in previous sections. In the first-principle calculations of Feng *et al.* (Feng et al., 2018), for monolayer MoS_2_ with lower Mo-vacancy concentration (4%), the imaginary part of the complex dielectric function has a slight blue shift, while for monolayer MoS_2_ with higher Mo-vacancy concentration (6.25 and 11%), the imaginary part of the complex dielectric function has a significant red shift. Our calculation results are consistent with the conclusion of Feng *et al.* that when Mo-vacancy concentration is high (6.26, 12.5, and 18.75%), the imaginary part of complex dielectric function of defective monolayer MoS_2_ appears obvious red shift. At low energy, V-Mo1 and V-Mo2-1 have an obvious transition peak at 0.93 eV, and V-Mo3 has a small transition peak at 1.56 eV. The red shift explains the dramatical improvement of the absorption in the NIR-II window, as shown in Figure 9B, where both V-Mo1 and V-Mo2-1 have an absorption peak at 1,270 nm. Considering the structure instability of V-Mo2-1 compared with the other two configurations of double Mo-vacancies, V-Mo1 may be a better choice for NIR-II applications.

#### Foreign Atom–Doped Structures

**FIGURE 10:**
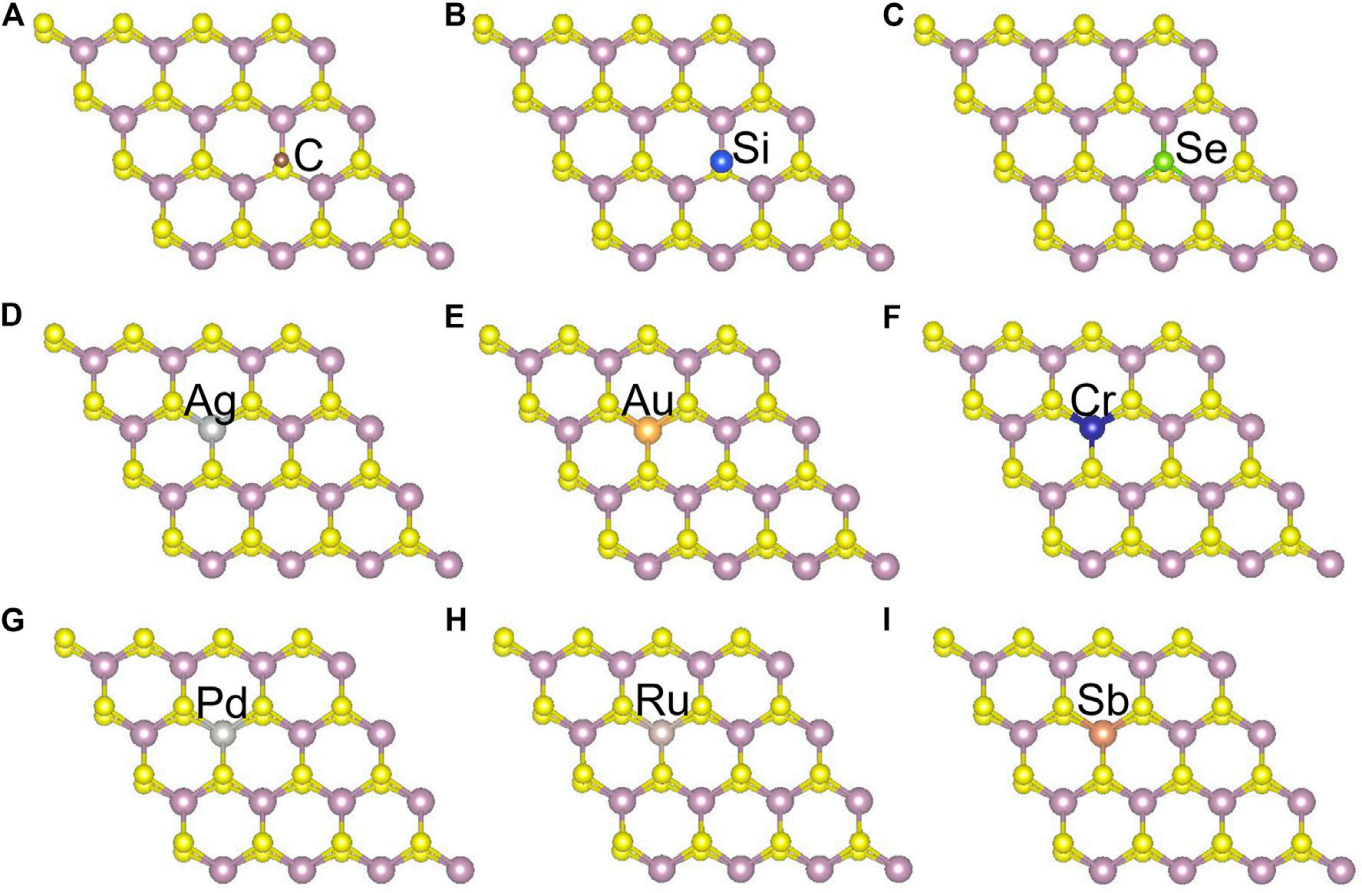
Monolayer 2H-MoS2 structure doped with different foreign atoms: **(A–C)** the substitution of a S atom with C, Si, and Se atoms, respectively; **(D–I)** the substitution of a Mo atom with Ag, Au, Cr, Pd, Ru, and Sb atoms, respectively.

**FIGURE 11:**
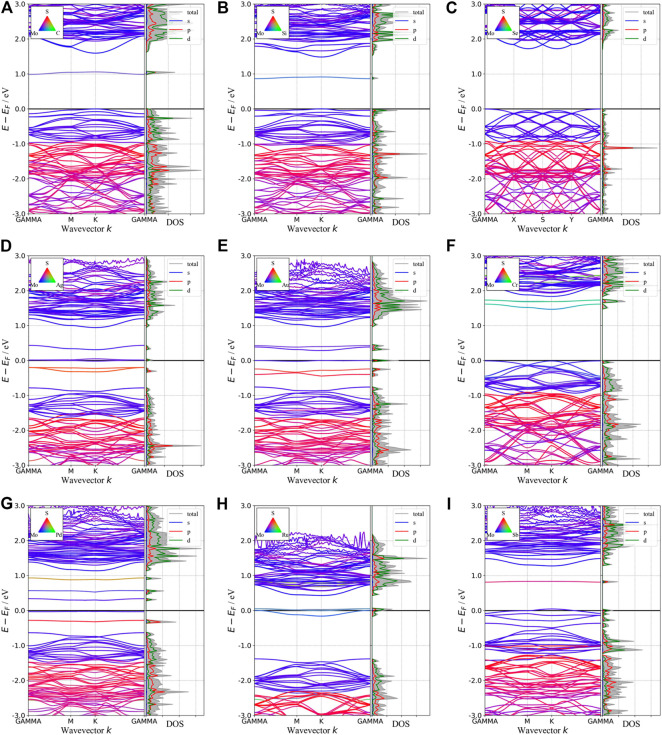
Band structures and pDOS of monolayer 2H-MoS_2_ after doping with different foreign atoms. The type of doped atoms is indicated by the color map in the upper left corner of each plot.

**FIGURE 12:**
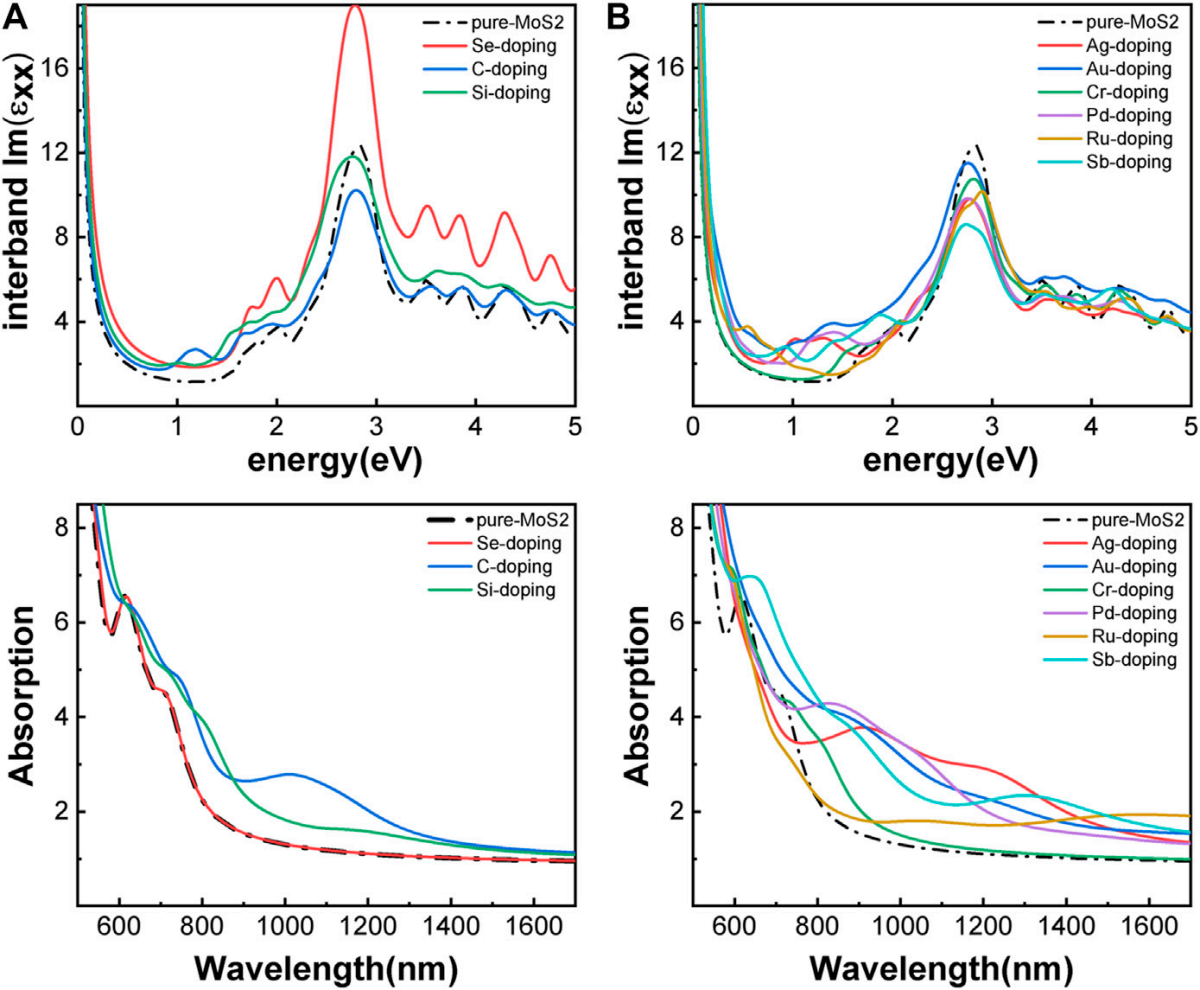
Optical properties of monolayer 2H-MoS_2_ after doping with different foreign atoms: **(A)** the imaginary part of the dynamic dielectric function; **(B)** absorption spectra. The left column is plotted for the substitution of S atom and the right for Mo atom. The black dotted line is for pure monolayer 2H-MoS_2_. The solid lines of different color refer to different doped atoms as shown in the legends.

Besides introducing vacancies, foreign atom doping is also a common method to manipulate the bandgap. As shown in Figure 10, we studied the substitution of atom S with C, Si, and Se and atom Mo with designed structures in which different elements replaced single S atom or single Mo atom with Ag, Au, Cr, Pd, Ru, and Sb. C, Si, and Se to replace S, respectively, and Ag, Au, Cr, Pd, Ru, and Sb to replace Mo, respectively. For dopants that fill single S-vacancy, C and Si have energy levels in the middle of the gap, while Se does not produce any localized states, as Komsa *et al.* expected in their work (Komsa et al., 2012). As shown in Figure 11, the doping of C and Si produces energy levels at 0.99 eV and 0.86 eV above the Fermi energy, respectively, and the doping of Se makes the bandgap increase to 1.73 eV, and our calculation result is in accordance with their findings. The doping of Ag, Au, Ru, and Sb turns the monolayer 2H-MoS_2_ into a metal. The doping of Cr and Ru leads to an impurity level within the original bandgap reduces the bandgap to 1.48 eV and 0.32 eV, respectively, which is consistent with those of Komsa et al., who observed vacancies filling and discussed the prospects for electron beam–mediated doping of TMDs (Komsa et al., 2012). Figure 12 shows the dynamic dielectric function and the absorption spectra of the doped structures. For the S substitution, doped Se leads to a sharp increase in the transition peak of the imaginary part of the dielectric function at 2.81 eV, but there is basically no change in the absorption in the NIR-II window, while doped C presents a small absorption peak at 1,020 nm. As for the Mo substitution, only Ag shows some improvement in the NIR-II absorption. In general, doping with foreign atoms is not as effective as introducing vacancies in the respect of improving the NIR-II absorption of monolayer 2H-MoS_2_.

#### Formation Energy and Stiffness Matrix

**FIGURE 13:**
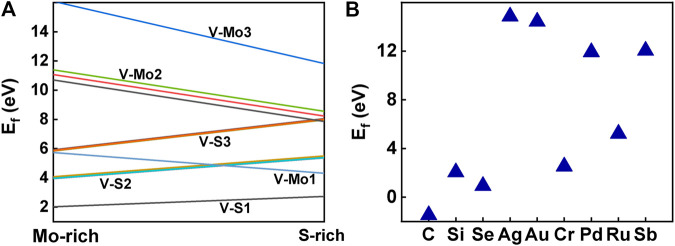
**(A)** Formation energies of different point defects, plot in Mo-rich and S-rich, which correspond to the chemical potential of its body-centered cubic bulk Mo and the S8 ring, respectively. **(B)** The doping formation energies of different foreign atoms.

Usually, the formation energy and the elastic stiffness constants are used to investigate the thermodynamic and mechanical stabilities of the system, respectively. To investigate the stability of the structure, we calculated the formation energy of all the structures with defects and those doping with foreign atoms, and the stiffness matrices of the three structures with defects that have good absorption performance in the NIR-II window, as shown in Figure 13. Considering that V-S1 has the lowest formation energy, it is the most common defect, which is consistent with the previous experiments (Zhou et al., 2013). Missing of the same number of S atoms is more stable than Mo atoms. Our calculations also show that the stability of the system becomes worse as the number of vacancies increases. In all the vacancies proposed in this study, single S, two S, and single Mo vacancies are relatively stable in either S-rich or Mo-rich environments. But Mo-vacancy is prone and easier to form under the condition of S-rich, and our calculation is the same as Feng’s conclusion (Feng et al., 2018). Combined with the ground-state energy, this indicates that V-S2-2, V-S2-6, and V-Mo1 are thermodynamically stable. As shown in Figure 13B, the foreign atoms doped on the monolayer MoS_2_ substrate are C, Si, and Se and Ag, Au, Cr, Pd, Ru, and Sb, respectively. However, in the case of Au, Ag, Pd, and Sb doping, substitutive doping has very high formation energy, so it is speculated that they may be more inclined to act as adsorbed atoms on the surface, which is consistent with the research of Wu *et al* (Wu et al., 2017). In addition, we found that the formation energy of different dopants substitution S-vacancy was lower than that substitution Mo-vacancy, which was consistent with our conclusion that the formation energy of Mo-vacancies was higher than that of S-vacancies.

In order to evaluate the mechanical stability of the point defects V-S2-2, V-S2-6, and V-Mo1, the elastic stiffness constants of the unit cell of the defects were calculated. For 2D materials, a necessary but not a sufficient condition for mechanical stability must be satisfied: $C_{66}>0$ and $C_{11}C_{22}-C_{12}^{2}>0$ (Maździarz, 2019; Imani Yengejeh et al., 2020). The calculated elastic stiffness constants of V-S2-2 are $C_{11}=69.66GP\text{a}$, $C_{22}=68.76GP\text{a}$, $C_{12}=16.58GP\text{a}$, and $C_{66}=26.15GP\text{a}$ , which satisfy the criterion of mechanical stability. In particular, $C_{12}\approx C_{21}$, this is due to the symmetric stiffness matrix. The elastic constants of V-S2-6 and V-Mo1 are $C_{11}=68.94GP\text{a}$, $C_{22}=69.05GP\text{a}$, $C_{12}=17.03GP\text{a}$, and $C_{66}=25.91GP\text{a}$ and $C_{11}=59.05GP\text{a}$, $C_{22}=58.98GP\text{a}$, $C_{12}=15.84GP\text{a}$, and$C_{66}=21.62GP\text{a}$, respectively. All of them satisfy the criterion of mechanical stability. According to the work by Akhter *et al.*, with increasing the doping and vacancy defect ratio, the elastic property of MoS_2_ film decreases, and when the defect ratio exceeds 10%, the elastic property decreases significantly (Akhter et al., 2020). It indicates that the structures in our work are mechanically stable because the proportion of defects under consideration is relatively low.

## Conclusion

In conclusion, based on the first-principle calculations, we found that monolayer 1T-MoS_2_ has a better performance in the NIR-II absorption than that of the 2H phase. However, monolayer 2H-MoS_2_ has the advantage of stability over the 1T phase, and its NIR-II absorption can be improved by introducing vacancies, while doping with foreign atoms is less effective. Our results show that there exist stable double-S-vacancy configurations (V-S2-2 and V-S2-6) and single-Mo-vacancy configuration (V-Mo1) that can be applied in the NIR-II absorption. Our calculations provide a useful guideline for the fabrication of monolayer MoS_2_ for the purpose of photothermal therapy guided by photoacoustic imaging.

## Methods

In this study, Vienna Ab initio Simulation Package (VASP) (Kresse and Furthmüller, 1996; Kresse and Furthmüller, 1996) has been used to calculate the electronic structure and optical properties of monolayer MoS_2_ films based on the projector augmented wave and Perdew–Burke–Ernzerhof (PAW-PBE) pseudopotential (Perdew et al., 1996; Kresse and Joubert, 1999). The numbers of valence electrons of each atom used in the calculation are listed in Table 2. The cutoff energy for plane wave expansion is set to be 500 eV. The generalized gradient approximation (GGA) is used for the exchange correlation energy functional to consider the nonuniformity of electron density. A vacuum layer of 15Å is taken in the *z* direction to avoid the interaction between the layers due to periodicity. The Brillouin zone integrations were carried out on the Γ-centered k-mesh (Monkhorst and Pack, 1976). For the 2H-phase monolayer MoS_2_ film, in order to better study the influence of different vacancy models and doping on its electronic structure, we first carried out 4×4×1 cell expansion on monolayer MoS_2_, which was a supercell with 48 atoms in total (16 Mo atoms, 32 S atoms). In the structural optimization, Monkhorst–Pack k-point meshes with size of 5×5×1 and 18×18×1 were used for supercell and primitive cell, respectively. The convergence standard of energy was 10^−6^ eV, and the convergence standard of force in the relaxation process was 0.02eV/Å. Ionic positions, cell volume, and cell shape were optimized by the conjugate gradient algorithm. In the self-consistent calculation, the method of Methfessel–Paxton (Gordienko and Filippov, 2009) order 1 is used in single-layer 1T phase MoS_2_, and the width of the smearing is 0.2, while the Gaussian smearing with the width of 0.05 is used for the single-layer 2H-MoS_2_. The tetrahedron method (Blöchl et al., 1994) with Blöchl corrections (use a Γ-centered k-mesh) was adopted in all the density-of-state calculations, and the width of the smearing was so small that it could be ignored. The (small) complex shift η in the Kramers–Kronig transformation is 0.1 when calculating the dielectric function.

**TABLE 2:** Valence electrons of the atoms used in the calculation.

Atom	Valence electrons	Configuration	Atom	Valence electrons	Configuration
Mo	14	4s^2^4p^6^4d^5^5s^1^	S	6	3d^2^3p^4^
Ag	11	4d^10^5s^1^	C	4	2s^2^2p^2^
Au	11	5d^10^6s^1^	Si	4	3s^2^3p^2^
Cr	12	3p^6^3d^5^4s^1^	Se	6	4s^2^4p^4^
Pd	10	4d^10^	–	–	–
Ru	14	4p^6^4d^7^5s^1^	–	–	–
Sb	5	5s^2^5p^3^	–	–	–

In addition, GW approximation (Shishkin and Kresse, 2006; Fuchs et al., 2007) is used to calculate the electron–electron scattering relaxation rate of 1T–metal phase monolayer MoS_2_. Combined with Drude-like intraband dielectric function contributions (Brendel and Bormann, 1992), the total dielectric function of 1T–metal phase MoS_2_ can be obtained. In the GW approximate calculation, the cutoff energy is set to be 300 eV, and 80eV is enough for the number of empty bands after the convergence test.

To investigate the formation of defects, the formation energy for defects can be calculated using the expression as follows (Persson et al., 2005):

$$E_{f}=E_{D}-E_{H}+\sum_{\alpha }n_{\alpha }\mu_{\alpha }\text{,}$$ (9)

where $E_{D}$ and $E_{H}$ are the total energies of the supercells with and without defects, respectively; $n_{\alpha }$ is the number of α atoms added ($n_{\alpha }<0$) or removed ($n_{\alpha }>0$); and $\mu_{\alpha }$ is the chemical potential of atom α. The chemical potential $\mu_{Mo}$ and $\mu_{S}$ satisfy the equation $\mu_{MoS_{2}}=\mu_{Mo}+2\mu_{S}$, where $\mu_{MoS_{2}}$ is the chemical potential of MoS_2_ unit in a pristine sheet. The formation for doped energies are defined as (Pan et al., 2015)

$$E_{f}=E\left(Mo_{16-m}S_{32-n}X_{m+n}\right)-E\left(Mo_{16}S_{32}\right)+mE\left(Mo\right)+nE\left(S\right)-\left(m+n\right)E\left(X\right)\text{,}$$ (10)

where m and n are the number of foreign-doped atoms, and $E\left(Mo_{16}S_{32}\right)$ and $E\left(Mo_{16-m}S_{32-n}X_{m+n}\right)$ represent the total energy of the system before and after doping. E(Mo), E(S), and E(X) are energy per atom for Mo, S, and foreign atoms X.

## Data Availability

The original contributions presented in the study are included in the article/Supplementary Material; further inquiries can be directed to the corresponding authors.
